# Sense-Making, Meaningfulness, and Instrumental Music Education

**DOI:** 10.3389/fpsyg.2020.00837

**Published:** 2020-05-05

**Authors:** Marissa Silverman

**Affiliations:** John J. Cali School of Music, Montclair State University, Montclair, NJ, United States

**Keywords:** sense-making, instrumental music teaching and learning, enaction and embodied cognition, eudaimonia (well being), meaningfulness and motivation

## Abstract

The purpose of this paper is to re-examine the nature of “meaning” and “meaningfulness” in the context of instrumental music education. By doing so, I propose to expand the ways in which instrumental music educators conceive their mission and the ways in which we may instill meaning in people’s lives. Traditionally, pursuits of philosophical deliberation have claimed that meaningfulness comes from either personal happiness (e.g., Jeremy Bentham and John Stuart Mill) or an impersonal sense of duty (e.g., St. Augustine, St. Thomas Aquinas, and Immanuel Kant). However, philosopher Wolf (2010) criticizes these positions in favor of a broader perspective, one that arises from understanding that there is a third sort of value, namely “meaningfulness.” Rightly understanding meaningfulness may help us engage more fully with a greater sense and understanding of the full potentials of eudaimonia: a life of significance and value for oneself and one’s community. Therefore, this paper links meaningfulness to a 4E (embodied, embedded, enacted, and extended) account of “sense-making” in/for instrumental music education. In doing so, I discuss the aims of public-school music education; aims that engage teachers and students in meaningfulness—a meaningfulness that is ethical, embodied, enacted, and extended—in, with, and through musics and, more directly, “instrumental” music making.

## Introduction

Traditionally, “instrumental music education” denotes the education of music through the learning and playing of musical instruments. While I am not going to argue with this prevailing assumption across our profession, I would like to expand our understanding of the natures and values of instrumental music education. Thus, what might be another way to interpret *instrumental* music education? Another adjectival meaning of “instrumental” connotes a helpful means to an oftentimes unforeseen end. In this context, I would like to ask, “instrumental” for what? For what might instruments be instrumental in helping students of all ages achieve?

The purpose of this paper is to investigate an integrated sense of “meaningfulness” both in life, more generally, and for instrumental music education, more specifically. Thus, this paper will proceed in four sections. First, I will briefly sketch out a 4E concept (embodied, embedded, enacted, and extended) of “sense-making.” Then, I will discuss meaningfulness. Next, I will relate sense-making to meaningfulness. I will conclude by linking sense-making and meaningfulness to instrumental music education.

Please note: While the quest for understanding the “meaning of life” is perennial and increasingly important, I will not attempt to address this question here. Instead, and following Wolf (2010, 2016), I examine what gives meaning *to* life. Asked differently, what is “meaningfulness” and why should instrumental music education care about this concept?

## Sense-Making

What does it mean to make “sense” of—to understand, feel, or engage with—something? Here, I would like to explore a 4E concept of “sense-making,” or a concept of mind that is embodied, embedded, enacted, and extended. Though before doing so, please note that “embodiment,” “embeddedness,” “enaction,” and “extension” are essentially “contested concepts.” Why contested? Because experts across varying fields (e.g., philosophy, neuroscience, psychology) have disparate views regarding each of these concepts. Thus, each concept warrants specific examination. However, due to the nature of this present philosophical investigation, I present a cursory sense of each, and, when needed, supply related literature that explores the individual components of a 4E concept more fully.

So, let us start with the nature of enaction, as “enaction” is the core of 4E cognition, and of being in the world. “The praxis of our living,” state Maturana and Varela (1992), is “enactive” (p. 241). In brief, what this means is that our understanding of our own existence comes from how we engage as selves and bring about ourselves in the world. Thus, as Stewart et al. (2010) explain, beings “enact” the worlds they live in; worlds and beings do not and cannot exist apart from one another; moreover, beings make it true that their “effective, embodied action in the world actually constitutes … perception and thereby grounds …[their] cognition” (p. viii). Note: Cognition is not “brain based,” but is, rather, enacted through a seamless set of processes and systems among the intersections of brain, body, and environment. Stated differently, to “enact” ourselves and our worlds, we put ourselves and our worlds “into existence through our action” (Gallagher and Lindgren, 2015, p. 392). In sum, as Gallagher and Lindgren (2015) write: “Cognition, as embodied and enactive, is not exclusively the result of neural processes in the head”; cognition is “accomplished in a dynamic set of interactions between brain and body, and between body and environment” (p. 394).

Following from this, and among numerous variables, people/agents are “coupled” to and with their surrounding worlds or “embedded” into contexts that contribute to a sense of ourselves, our place in the world, and give way to each and every motivation that propels us forward in our lives. Additionally, our “coupling” speaks to the dynamic, intersubjective nature of self and of an embodied perception (Fuchs and De Jaegher, 2009).

“Embodiment” refers to the many ways in which our corporeality determines our place in and sense of the worlds in which we live. Because we are “actors” (Gallagher and Lindgren, 2015) —and because we enact our sense of self and world—we are feelingful, embodied beings. Without our bodies and our embodied involvement in time and place, we can have no real sense—literally and metaphorically—of ourselves and others. A 4E concept of engaging in the world is used, here, to emphasize the ways embodiment, and the ways in which human beings are simultaneously autonomous and interconnected—through feeling-and-knowing—to our contexts and worlds (e.g., Varela et al., 1991; Thompson, 2007; Di Paolo et al., 2010; van der Schyff and Krueger, 2019).

Importantly, and by way of explaining “extension,” Noë (2009) states our brain, body, and the environments we participate within not only complement each other, but, moreover, enact our sense of self, the world, and our intimate, interconnectedness to the worlds in which belong. Now that some of the individual components of a 4E concept are illustrated, what is meant by “sense-making”?

Noë’s concept of perception, cognition, and embodiment as brain-body-world is foundational for any 4E approach to sense-making. By “sense-making,” I am exploring both the literal—i.e., as in making sense of something or someone—and the physical—i.e., as in feeling-through a stimulus of any kind. Therefore, in line with both Noë (2009) and Gallagher (2017), an enactive perspective on sense-making acknowledges that human beings have evolved the ways they have because we possess a brain in a body that is not isolated in the world. Instead, the brain is “dynamically coupled to a body that is dynamically coupled to an environment” (Gallagher, 2017, p. 115). Further, the “organism”—or the brain-body-mind system—is “operating within the situation itself rather than on a model of the situation inferred by the brain” (p. 115). This thesis notes that such a coupling of brain-body-environment comes to be because of and through the very nature of our “neuronal processes, bodily movements, affects, anatomy and function, and environmental regularities” (Gallagher, 2017, p. 115).

It is essential that, at this point, we note predecessors of any 4E concept of sense-making. Poincaré’s (1907) research on spatial perception, Piaget’s (1936) work on sensorimotor equilibrium, and Goldstein’s (1934/1995) investigations of self-actualization all point to a 4E concept of sense-making.

Philosophers, too, contribute to a 4E concept of sense-making. For example, *Experience and Nature* (Dewey’s, 1925/1958) examines our embodied human experiences both in and of the worlds in which we engage. Thus, 4E principles are foreshadowed in Dewey’s investigation of our worldly “transactions^1^”—where body, mind, and world come together to form an interconnectedness of meaning-making potentials. Notably, in his analysis of Dewey’s connections to enactive principles, Gallagher (2017) explains that Dewey’s (1894, 1895) early theories on emotion emphasized two points worth mentioning: first, emotion *is* action; second, “emotions are not reducible to a set of bodily states since the body is always coupled to an environment, and always includes situational aspects” (p. 63). Other philosophical roots of a 4E concept of sense-making trace to the phenomenology of Heidegger (1962, 1968, 1969; specifically, and among other aspects, Heidegger’s “being in the world” and pragmatist perspectives of our environments and its tools being “ready-to-hand”), Husserl (1960, 1980, 1989; specifically, and among other aspects, Husserl’s investigation of “perception” of the environment and its tools and the potentials found therein), and Merleau-Ponty (1948/2004, 1962, 1964; specifically, and among other aspects, Merleau-Ponty’s sense of “embodiment,” which is somewhat explored later in this paper).

Contemporary research within the sciences—see the work of Hutchins (1995) and Beer (2003)—have important compatibilities with a 4E approach. While research of Damasio (1994, 2009, 2010), Clark (1997, 2006)^2^, Hurley (2002), LeDoux (2002, 2019), and Prinz (2012) does not necessarily utilize the combined terms “embodied, embedded, enacted, and extended,” important principles of 4E sense-making underlie their research. Di Paolo et al. (2010)—with their enactive concept of the mind (for discussions about their work in relation to music and music education, see e.g., Silverman, 2012; van der Schyff, 2013)—also examine the ways in which we are embodied, embedded, enacted, and extended beings.

Within the field of philosophy, Noë (2004) and Thompson (2007) state, perception and consciousness and, therefore, sense-making, all happen because of the numerous interactions among brain, body, and world (Silverman, 2012; Elliott and Silverman, 2015). Without these components and interactions, consciousness cannot occur, nor does it exist (e.g., Noë, 2004; Thompson, 2007). And without consciousness there is no sense of sense-making. Furthermore, and most importantly according to Thompson (2007), no enactment of self.

A theme shared by all these scholars needs special emphasis because it shatters a longstanding dualism, which in turn blocks a fuller understanding of “self” or “personhood.” That theme is: A being that possesses cognition does not exist “out there” solely in the world. Instead, a being that possesses cognition is, as Thompson (2007) states, “*a relational domain enacted or brought forth by that being’s autonomous agency and mode of coupling with the environment*” (cited in Silverman, 2012, p. 99; italics mine). Therefore, and stated differently, cognition occurs because one’s personhood is not only interconnected, relational, and essentially enmeshed with the worlds that one inhabits; cognition and personhood are possible *because* of this coupling between self and world (see Gallagher, 2005, 2017; Thompson, 2007).

Similarly, and in line with these above-mentioned scholarly predecessors, van der Schyff and Krueger (2019) examine a 4E account of the mind, perception, and, therefore, of identity formation. They explain that sense-making involves more than just mental and emotional processes, but also engages bodily and environmental factors (see also, Noë, 2004, 2009; Thompson and Stapleton, 2009; Gallagher, 2017). As Krueger (2018) states:

we routinely “offload” our thinking onto body and world. Tilting our head to make sense of a rotated image or text, for example — instead of rotating an internal representation — reduces information-processing demands …. Similarly, we use gesture to represent solutions to mathematical problems … sketchpads to scaffold artistic creation …, models to better understand scientific theories …, and smartphones, search engines, and cultural institutions to support memory…. These beyond-the-head targets of our offloading generate ongoing feedback loops that transform our cognitive profile in real-time and, in so doing, help us negotiate complex cognitive tasks. From a 4E perspective, understanding how minds work requires looking beyond heads. And for a 4E view called the “extended mind thesis,” these materially scaffolded feedback loops are so important for driving thought and experience that they should be seen as part of the (extended) mind itself. (p. 1)

All these integrated components—or a 4E concept of sense-making—provide important considerations in understanding human consciousness and personhood. A 4E concept of sense-making is essential to all we think, believe, value, and do—including music making. However, before we can conclude anything further about determining value for ourselves, let us first consider the nature of “meaningfulness.”

## Meaningfulness

As alluded to at the outset of this paper, to possess “meaning” is not necessarily the same as something being “meaningful” (James, 2010; Wolf, 2010; Metz, 2011; Thomas, 2019). Still, examinations of both the “meaning of life” and “meaningfulness in life” fall under the domain of fields related to practical reasoning and ethics. In Western philosophical traditions, attempting to understand the “meaning of life” and “meaningfulness in life” is perennial and dates back to ancient Greece. Even though Williams (1981) notes there are sometimes short-sighted attempts to answer these increasingly important questions, we owe it to ourselves to continue to attempt to think through what “meaning in life” is and why thinking about this matters.

Still, while Socrates’ axiom is that “the unexamined life is not worth living,” both Williams (1981, 1983, 2012/1993) and Wolf (1982, 2010) point out that we may lead meaningful lives without giving much thought to conceptualizing where that “meaning” may come from (Silverman, 2013; Elliott, 2020). Indeed, the “desires” and motivations that provoke, inspire, and “propel one forward do not have to be very evident to consciousness, let alone grand or large; one good testimony to one’s existence having a point is that the question of its point does not arise, and the propelling concerns may be of a relatively everyday kind such as certainly provide the ground of many sorts of happiness” (Williams, 1981, p. 11).

Regardless, as professionals, we must consider what makes life, music, music teaching and learning, and instrumental music education “meaningful” if, for no other reason, than to well understand the nature and values of our work (Elliott and Silverman, 2015). So, what is meant here by “meaningfulness”? Wolf (2010, 2016) examines “meaningfulness” by comparing and contrasting two prominent positions within the field of ethics: the Fulfillment View and the Larger-than-oneself View. The Fulfillment View suggests that we experience meaning when we are fulfilled. The Larger-than-oneself View, notes Wolf, recognizes we experience meaning when we find ourselves serving the needs of that which is beyond ourself. Both viewpoints, state Wolf, are critical components in understanding the motivations of people with that which tends to fuel our lives.

However, according to Wolf (2010), while human motivation is categorized in one of two ways, egoistic (and self-interested) or altruistic (and moral), such motives may not fully capture the reasons for what propels us in our lives. As Wolf (2016) states, motivations that may not be egoistic or altruistic “are neither peripheral nor eccentric. To the contrary, they are the reasons and motives that engage us in the activities that make our lives worth living” (p. 254). So, we might ask: What kind of motivations are neither egoistic or altruistic, and what is the “distinctive character” (Wolf, 2016) of such motivations that challenge this simplistic dualism?

James (2010), Wolf (2010, 2016), Metz (2011), and Thomas (2019) all seem to maintain that “meaningfulness” may come about when we love people, objects, and/or activities that are worthy of love. Wolf (2010, 2016) provides specific examples such as visiting relatives who would feel better being visited, helping friends relocate, as well as making her daughter’s Halloween costume. In each instance, Wolf (2010) notes: “I act neither out of self-interest nor out of duty or any other sort of impersonal or impartial reason. Rather, I act out of love” (p. 26).

Again, and for emphasis given where we are headed with this paper, “reasons of love” (Wolf fittingly utilizes Harry Frankfurt’s terminology) move us to purposefully engage in a variety of activities and interests that fill our lives (Silverman, 2013). In fact, and further, we may act from reasons of love for which “reason” (i.e., logic, contemplation, and/or conceptualization) has very little business. We do not need to notice the value that something, someone, or some activity possesses prior to having “reasons of love” for which meaningfulness is the result (Frankfurt, 2004). Oftentimes, we act meaningfully before even noticing how or in what ways our pursuits seem meaningful.

Please note: Wolf does not suggest we remove the egoistic or moral activities and interests from our lives. What she is suggesting is we might benefit from recognizing whether such activities and interests provide meaningfulness for us. In other words, if we focus on meaningfulness instead of self-fulfillment or altruism, we may find we possess much more meaning in our lives. Or, we may find we could use more meaning for ourselves in our everyday lives.

So, when I am called to perform challenging “new” (for me) music, I often “over prepare.” Something similar happens when I plant and weed the tomatoes, basil, and flowers on the balcony of my apartment (even though the height of my balcony bothers me); or when I attempt to photograph a butterfly who just landed on a flower (as frustrating as this may be, given butterflies tend not to sit still for photographs); or when I purchase and wrap birthday presents for those people who are important to me. What motivates me to practice, garden, take pictures, or seek out the perfect present exists *outside* myself (Wolf, 2010, 2016; Silverman, 2013). What draws me to do these things is love. I do not care if these activities are “good for me.” Aesthetic theories of art suggest that I practice for “music’s sake” or take pictures for “art’s sake” (e.g., Wimsatt and Beardsley, 1958; Scruton, 1999; Reimer, 2003). But, as Carroll (2010, p. 86) states, “doing something for its own sake” is as unfounded as it is simplistic; Wolf (2016) notes believing this is equally “misleading and obscure as well as pretentious” (p. 255). Rather, “reasons of love” motivate me to engage in these pursuits (Wolf, 2010, 2016; Silverman, 2013). Thus, states Wolf (2016), “reasons of love”—whether of people, ideals, or other sorts of objects—have a distinctive and important role in our lives,” all which have little to do with “self-interest” or “morality” (p. 255).

Wolf (2016) is keen to point out that, just because we are motivated by “reasons of love,” such motives may not justify potential outcomes. Might I “scare away” the butterfly I am attempting to photograph? Or, could I mis-interpret the piece of new music I’m learning? And might I, inadvertently, pull out some of the roots of my tomato plants while attempting to rid them of weeds? Indeed, says Wolf, and, moreover, “the energy and attention” I may give to the activities, objects, or persons “may be disproportionate” to what is warranted by the activities, objects, or persons (p. 255). And in such instances, claims Wolf, such actions on my part will not eventuate in meaningfulness because the “worthiness” of my behavior is not independent of my own agenda.

Markedly, meaningfulness comes about “when subjective attraction meets objective attractiveness, and one is able to do something about it” (Wolf, 2016, p. 261). Additionally, says Wolf, value, and therefore meaningfulness, occurs only when one is “actively (and lovingly) engaged” and when there is “objective worth” in the pursuits one finds valuable. This view, or the “Fitting Fulfillment View,” is not meaningful says Wolf (2016) because of the ways we *feel* when so engaged. Instead, we find our lives meaningful because we are interested in “being” the kind of person who loves activities, objects, and persons that are worthy of our attention, dedication, and energies:

We do not satisfy those interests simply by thinking or feeling that they are satisfied any more than we satisfy our interest in not being alone simply by thinking or feeling that we are not alone. To have a life that not just seems meaningful but is meaningful, the objective aspect is as important as the subjective. (Wolf, 2016, p. 263).

At this point, a reader may be suspicious of such a perspective about meaningfulness. Thankfully, Wolf (2010, 2016) is careful to explain the potential issues inherent with her thesis. One of these is to ask: Is there really such a thing as “objective value”? To challenge readers’ suspicion, and instead of asking this question, she proposes we ask: “Which projects are valuable?” and “Which activities are worthwhile?” (p. 264). These questions are free for each and all to ask and answer. Thus, she states:

I assume that we will answer them better if we pool our information, our experience, and our thoughts. Presumably, the task of determining which activities and projects are worthwhile is a never-ending process, both because, as fallible creatures, our judgments of value will always be somewhat tentative, and because at some level the sorts of things that have value are apt to change over time. The absence of a final authority on the question of which things have value, however, does not call into doubt the legitimacy or coherence of the question itself or of the enterprise of trying to find a more or less reasonable, if also partial, tentative, and impermanent answer. (Wolf, 2016, p. 264)

Why is this the case? We find value in objects, activities, and people because we are social beings who engage in social practices toward “some kind of practical end” (Elliott and Silverman, 2015, p. 51). In the case of my flute playing, gardening, or photography, all are social practices that showcase the integration of people, processes, products, and contexts. Related to this, we find that the human “goods” or values—or meaningfulness—are determined “by and for the people who are involved” within each social practice “itself” (Elliott and Silverman, 2015, p. 51).

By looking at how and why certain types of project or activity provide meaning for the people who engage in them (and also perhaps at what can interfere with these activities’ potential for meaning) we can better understand these activities and what we get from them without making comparative or universal judgments. We can perhaps also better design such activities and projects in a way that is likely to enhance their potential meaning for others. (Wolf, 2016, p. 268)

## Sense-Making, Meaningfulness, and Instrumental Music Education

In many respects, meaningfulness and sense-making share common thrusts. Notably, meaningfulness as related to sense-making casts light on how we interpret the things in our life that give us a sense of “purpose” as well as that which we deem valuable for our overall flourishing. Such purpose is embodied, embedded, enacted, and extended, whether or not we “verbalize” or “conceptualize” these pursuits. How so? As Thompson and Stapleton (2009) write:

even the simplest organisms regulate their interactions with the world in such a way that they transform the world into a place of salience, meaning, and value—into an environment (*Umwelt*) in the proper biological sense of the term. This transformation of the world into an environment happens through the organism’s sense-making activity. (p. 25)

Thus, one of the main aims of sense-making is defining value—whether consciously or non-consciously—for the agents within their social worlds and contexts. In discussing the “meaning” of meaningfulness, or, rather, meaninglessness, Eagleton (2007) notes that people who feel that their lives “lack meaning” are actually expressing their “lives lack significance.” What this points out, says Eagleton, is such lives “lack point, substance, purpose, quality, value, and direction” (p. 64). So, as Thomas (2019) delineates, “when someone desires a life which is meaningful, they are essentially hoping for a life which is sense-full or full-of-sense” (p. 1558).

Relatedly, as already explored above, Wolf (2010, 2016) maintains that meaningfulness connects us to place, persons, and projects of love; such love indicates “value.” What does this mean for instrumental music making and, therefore, *instrumental* music education? In *Music Matters* (Elliott and Silverman, 2015), David Elliott and I examine important dimensions of personhood, and some of the numerous ways in which we bring our self-other worlds into holistic being, and, moreover, the many ways music and music education matter. There, we make a case for—without then stating so—a 4E perspective of sense-making, meaningfulness, and music and music education:

the holistic nature of *you* is *always greater than the sum of your unified processes*. These are always in a continuous state of modification and change. So personhood includes but is *not* limited to self-awareness, self-identity, spirituality (and more) that emerge from your intrapersonal and interpersonal interactions with your socially situated communities, norms, and values. All dimensions of personhood make all others possible. Take one part away and personhood will be impaired to some degree or another. Your unified nature is what enables and powers the you-ness of you as an extraordinarily complex being that experiences everyday life as a seamless flow of conscious and non-conscious experiences of all kinds (thoughts, intuitions, emotions, sensations, memories, etc.). (italics in original; p. 157)

We examine, moreover, the ways in which we are complex beings; beings who come to be because we are “*completely unified* (embodied), *interdependent*, and *actively engaged in enacting or bringing about our self-other worlds into being*” (pp. 156–157). So, personhood is not located within us in a singular way. Instead, persons emerge—and understand themselves as persons—and are enacted because of the “dynamic syntheses” of our many “embodied processes” that are in/of our worlds. Because of this, we explore some of the many swirling ways—e.g., attention, perception, cognition, emotion, memory, volition, and/or conscious-and-non-conscious states of experience—that, in combination, make up our embodied, embedded, enacted, and extended account of sense-making and, therefore, “personhood” (Elliott and Silverman, 2015).

Importantly, our personhood comes about because of our body mapping abilities, which are inclusive of, among other aspects, mirror neurons (Elliott and Silverman, 2015). For present purposes, we might ask why and how is it that a “body comes to mind” (Damasio and Damasio, 2006, p. 16; Johnson, 2007) when frightened, or hungry, or feeling joy or “chills” during a powerful musical experience, or when having bodily experiences of singing, blowing air through a flute, or strumming a guitar (Elliott and Silverman, 2012, 2015; also see, Bowman, 2004; Bowman and Powell, 2007)? Along with other researchers, Damasio and Damasio (2006) contend that in addition to chemical and neural signals and multiple personhood systems that flow both to and from the brain, we have a biological scaffolding system—or, stated differently, body mapping—that affords our “minding the body” and “em-bodying the mind” in a dynamic dance, as two partners would dance across the world with each other (Damasio and Damasio, 2006; Elliott and Silverman, 2015).

Please note: while Damasio’s research primarily utilizes “brain-centered” terminology, limiting Damasio’s work to the brain’s neuronal networks as they engage the body would diminish its scope. As Damasio (1994, 1999, 2010) examines, the body is intimately involved in all sorts of conscious and non-conscious processes; we might refer to such bodily involvement as “background feelings.” Moreover, Damasio (1994) explains that consciousness can be understood as “the feeling of life itself, the sense of being” (p. 150). In other words, Damasio’s (1999, 2010) research acknowledges that consciousness is “the feeling of what happens” or the “feeling of what it is like to be oneself.” And while the brain is essential in our “minding our body,” our “bodies” are the “vessels” in which we are interconnected to our worlds.

Returning to body mapping, most of our body maps are located in the brain’s cerebral cortex. For example, the primary visceral map is your body-brain-mind’s way of creating a representation of your internal processes: “This map is uniquely super-developed in the human species, and it gives you a level of access to the ebb and flow of your internal sensations unequaled anywhere else in the animal kingdom” (Damasio and Damasio, 2006, p. 17). This mapping system captures a huge range of our body states and environmental-world-context experiences that occur moment to moment, which cause us to experience continuous changes in everything we think and do.

Thus, when a flute player “cracks” a note while playing a particularly high-register passage, or a violinist uses a bowing pattern that does not suit a melody she is interpreting, the embodied, embedded, enacted, and extended mind—depending on levels of experience—works to help readjust things. Also, our body-mapping systems continuously update our maps in relation to everything we see, hear, taste, touch, think, feel, intuit, sense, and otherwise experience (Elliott and Silverman, 2015).

Relatedly, body maps are plastic; they shift, modify, and mature over a lifetime (Blakeslee and Blakeslee, 2007, p. 1). Thus, as anthropologists tell us that, because our bodies are not only biological, but also ecological, phenomenological, social, cultural, gendered, and conjectural (at the very least), cultures have their own ways of knowing-through-the-senses and, therefore, engendering and enculturating people’s body maps (Elliott and Silverman, 2015; see also Johnson, 2007). Varying with the culture, then, a person’s senses of “sight, sound, touch, taste, smell, balance, and personal space are all mapped differently” (Blakeslee and Blakeslee, 2007, pp. 207–208). The same thing applies to musical sounds, felt musicing and listening, and musical-emotional experiences (Krueger, 2009, 2018; Elliott and Silverman, 2015).

Additionally, body maps function to create a personal space around us (technically termed “peripersonal space”), which extends outwardly depending upon the “tools” we typically use (Blakeslee and Blakeslee, 2007, p. 127). Through special mapping procedures, our brains extend and add this space to our limbs and body: “In each social context, you choose your comfort zone and broadcast it to others with body language, eye contact, posture, facial expression, and how you listen” (Blakeslee and Blakeslee, 2007, pp. 127–128). When objects and people enter your domain and peripersonal space, specific brain cells and networks fire up. So, physiologically, we have our own “personal bubbles” that surround us wherever we go; they inform us, not only about a vast range of details in our world-environments-contexts, but also about our potential to perform actions in or enact—e.g., to bring to mind, consciousness, empathy—our own spaces.

One consequence of this extraordinary human attribute is that the objects, artifacts, tools, and technologies we use frequently in our daily lives—whether musical instruments, computer keyboards, social media, bicycles, automobiles—feel like extensions of or natural parts of our personal selves, especially when we use them often and become skilled in their use (Elliott and Silverman, 2015). Understanding our use of tools in this way gives more fuel to the claim that “you are what you do.” Such doing is not only embodied in that it is intimately felt, it informs who we are and hope to become. Our identities are shaped and reshaped depending on the habits we form, the embodied engagements we produce, and the ways in which tools are associated with our thinking-knowing-doing.

So, when a person plays, say, the cello frequently, her cello and bow feel like a part and extension of her body. Consequently, and due to the nature of specific instruments, a trombone player’s peripersonal space is mapped very differently than a piccolo player’s peripersonal space. As Alperson (2008) notes: “Many musicians put the matter clearly when they speak of their instruments as extensions of their bodies. The truth is that it is difficult to say where the instrument ends and the rest of the body begins. In this sense, musical instruments are embodied entities” (p. 40).

Now it makes sense to discuss the subtle difference between a “tool”—like the musical instrument of a music maker—being either an “extension” of, or “incorporated” into, a person’s peripersonal space. Consider Merleau-Ponty’s (1962) famous case of the blind man and his cane. I quote it here at length for emphasis:

The blind man’s stick has ceased to be an object for him, and is no longer perceived for itself; its point has become an area of sensitivity, extending the scope and active radius of touch, and providing a parallel to sight. In the exploration of things, the length of the stick does not enter expressly as a middle term: the blind man is rather aware of it through the position of objects than the position of objects through it. The position of things is immediately given through the extent of the reach which carries him to it, which comprises besides the arm’s own reach the stick’s range of action. The points in space do not stand out as objective positions in relation to the objective position occupied by our body; they mark, in our vicinity, the varying range of our aims and our gestures. To get used to … a stick is to be transplanted into [it], or conversely, to incorporate [it] into the bulk of our own body. (p. 143)

As Merleau-Ponty points out, the man who is adept at utilizing a cane to “extend” his reach is, in fact, not merely extending his peripersonal space, but is, in fact, incorporating the cane into his body’s schema. Additionally, the cane is no longer an object “out there” in space for use; it has become a part of his personhood.

So, continuing to follow Merleau-Ponty who discusses an organ and an organist, musical instruments, in the “hands” of those who habitually use them—or, in the habitus of our personhood processes—take on similar, if not a further “incorporated” significance. How? As Tanaka and Donnarumma (2019) explain, the organist knows, in an embodied way, the various mechanisms of an organ: its pedals, pulls, and stops. While musicing, “she incorporates” how the mechanics of the organ—especially one she knows well^3^ —can allow her to “achieve given musical and emotional values” (p. 82). The organist’s “gestures draw ‘affective vectors’ mediating the expressiveness of the organ through her body. The organist does not perform in an objective space, but rather in an affective one” (p. 82). This kind of embodied epistemology is the basis of a musicer’s enactive knowing, thinking, and doing (Tanaka and Donnarumma, 2019; see also, Varela et al., 1991; Di Paolo et al., 2010; van der Schyff, 2013; Matyja and Schiavio, 2013; Elliott and Silverman, 2015; van der Schyff and Krueger, 2019).

Still, perhaps the distinction between “extension” or “incorporation” related to a body’s use of a musical instrument needs further refinement. For this, let us turn to Thompson and Stapleton (2009) and their discussion of the “body-as-object” and the “body-as-subject” (p. 29). Drawing from the work of Legrand (2006, 2007) and Thompson and Stapleton (2009) state:

The body-as-object is the body perceived and recognized as mine (e.g., “These are my hands.”). The body-as-subject is a structure of experience; it is that through which the world is experienced. As such, the body-as-subject is transparent: “The body is transparent in the sense that one looks through it to the world. At this level, pre-reflective bodily experience is precisely the experience of the world as given through the ‘transparent body.’ The latter is not perceived as an object but experienced specifically as a subject perceiving and acting, that is, in-the-world” (Legrand, 2007, p. 504 cited; p. 29).

As Thompson and Stapleton note, tools and resources—and, yes, musical instruments—are “incorporated” by the body and acquire this “transparency” (p. 29). A musicer’s musical instrument is no longer an “object” outside of the body, thereby extending the body’s “reach.” Instead, the musicer experiences the world through the instrument; the instrument, following Thompson and Stapleton, “has become transparent” (p. 29). What does this mean for personhood and therefore sense-making? They sum up by stating that for something external to a person’s body to become part of one’s “cognitive system”—even more, I would argue, as metacognitively part of her sense of personhood—the external entity “must function transparently in the body’s sense-making interactions with the environment” (p. 29). They continue by stating “that tools and aids that conform to transparency are incorporated into the neurophysiological body schema” (p. 29).

What might the above mean for instrumental music education? Hopefully, some conclusions may seem self-evident at this point, namely that instrumental music education is *instrumental* in expanding the potentials of students’ personhood. In expanding students’ personhood, students’ potentials are, too, expanded. And, instrumental music students-as-music makers expand their worlds—both metaphorically and quite literally—by increasing “the reach” of their peripersonal space, body maps, and embodied capacities and capabilities (to varying degrees).

Another important conclusion is that instrumental music teaching and learning should be guided by a philosophy of music education that embraces 4E understandings of a person’s sense of self and personhood (van der Schyff, 2013). A philosophy of music education such as the one explored in *Music Matters* (Elliott, 1995; Elliott and Silverman, 2015) has the potential to provide logical foundations and pragmatic principles for ensuring that the facilitation of people’s sense-making (musical and otherwise) and personal identity development is ethical and beneficial in many ways. Thus, a praxial orientation focuses on:

• The why–what–how–where–when of effective, democratic, and ethical informal and/or formal education in, about, and through all forms of music-making—including performing, improvising, composing, arranging, conducting/leading, music and dancing, musical therapeutic participations, etc.
• Empowering people to make and listen to music with an “ethic of care”—care for individuals and their communities. (Elliott and Silverman, 2015)

Music education and, therefore, instrumental teaching and learning, should be guided by musical mentors’ informed and ethical dispositions to “act truly and rightly” with continuous concern for protecting and advancing meaningfulness, human flourishing, and wellbeing (Elliott and Silverman, 2015; Smith and Silverman, 2020). From this viewpoint, musical mentors who are *solely* concerned with teaching instrumental musicing techniques are not engaged in *praxis*—ethical knowing, thinking, and doing—and praxial musical personhood formation. To promote a socially constructive and ethical musical personhood, musical mentors of all kinds must harness musical affordances with a conscious commitment to an “ethic of care” and care-guided actions.

A central humanistic purpose of musical involvements and instrumental teaching and learning is to pursue what Aristotle and many other philosophers consider the highest human values: a “good” life of flourishing, wellbeing, fellowship, meaningfulness, virtue, and happiness for the benefit of oneself and others. All these values can be summarized by one word: *eudaimonia*. If we conceptualize and engage with musics as musical praxes—a “move” that emphasizes the critically reflective and ethical dimensions of instrumental musicing and listening—all forms of ethically minded music-making offer potential opportunities for pursuing eudaimonia. In addition, if mentors examine and guide musicing and musical personhood formation in relation to praxial concepts and eudaimonia, it is more likely that musical and personal identity construction will become central aims of informal and formal school and community music programs.

## Conclusion

Why should we consider a 4E foundation of sense-making in relation to instrumental music education? Primarily, and conceptually, unless we understand the multi-layered nature of personhood and all it entails, we run the risk of lessening the importance and intricacies of all that goes into everything we say, think, believe, do, value, and create. And because in many respects sense-making is integral to being, all we value—whether that “value” be egoistic, altruistic, or from some other perspective—stems from our personhood as embodied, embedded, enacted, and extended.

Additionally, we might consider that, while egoism and altruism are typically deemed as opposites, this assumption rests on a premise that does not fully consider a being’s embodied, embedded, enacted, and extended personhood. Indeed, as John Donne pointed out, “no man is an island entirely unto himself”; we are interconnected to each other and to our worlds, and our sense of self happens because of our interconnectedness (Pettersen, 2011). Because of this, how we understand our selves and our worlds come from 4E sense-making. Relatedly, and because we are embodied, embedded, enacted, and extended beings with a personhood, our values (e.g., including the habitus of meaningfulness), too, are interconnected to our worlds around us. Thus, meaningfulness shares many commonalities with care and care-fullness (Gilligan, 1982). How? Because when we find meaning in our lives, we (consciously or non-consciously) connect to persons, objects, and projects that grab hold of our care, commitment, and love.

Meaningfulness takes on new dimensions when considered from 4E perspectives: it, too, is embodied, embedded, enacted, and extended. Its subjective, objective, and intersubjective dimensions come about because *we make it true* that such reasons of love are important because of the ways in which those persons, objects, and projects connect us both to ourselves and our worlds in significant ways. Please note and by way of reminder: Such value-creating as “meaningfulness” need not be a conscious deliberation. In fact, meaningfulness may not be recognized until after the persons, objects, and projects are already engaged or enacted.

Following from this, sense-making and meaningfulness take on new perspectives when we consider the ways in which activities, persons, and, yes, even instruments matter—to both our sense of personhood and as ways we engage with/in/through projects of love with the world. Why? Because as instrumentalists, and through instrumental music education, people who learn to perform, improvise, and compose (with new media or conventional instruments and vocal ensembles), and who thereby participate in the caring for, maintenance, fluidity, reinvention, and advancement of various instrumental music praxes, the people of those praxes gain yet another internal good. They achieve what MacIntyre (1984) calls a certain kind of life (p. 190; see also, Sparshott, 1988). Importantly, engaging in instrumental musicing and listening ethically adds meaningfulness to the content and continuousness of one’s sense-making, and therefore, one’s life in the world. By entering into, learning, and expanding musical praxes through living a part of one’s life as a musicer—whether amateur or beyond—a student (child through adult) gains a valued way of being and becoming: she gains the unique value of living out a greater or lesser part of her life as a maker of musics (MacIntyre, 1984, p. 190). Summarily, when instrumental music students enter into musical praxes as active, reflective practitioners, they not only potentially develop “a certain kind of life” through musics (Regelski, 2005, 2016; Elliott and Silverman, 2015). Their teachers also create conditions for a sustained development of their students’ personhood.

Thus, learning to be an instrumental musicer of a reasonable range of musical praxes is one of several viable educational ends for all people. Consider the many musical activities one can pursue as an instrumentalist, depending upon the musical-social practices involved: performing, composing, improvising, arranging, conducting/leading, recording, producing, musicing and dancing/moving, musicing and worshiping, and more. An instrumentalist can become a coach and teach one or more musics to others, whether formally or informally. An instrumentalist can write about music, lecture about music, collect artifacts that surround musical ways of being (e.g., recordings, letters from famous instrumentalists), read about music, discuss music, argue about music, and so forth. And as a listener of music making—whether as an instrumentalist or not—one can “maintain the fabric that connects one to others” (Wolf, 2010, p. 130), knowing that a “bond exists” between self and others in intimate ways, especially given’s music’s (emotional, social, cultural) potencies (Elliott and Silverman, 2015).

In many ways, we can draw similarities between the above and Noë’s (2015) examination in *Strange Tools* of organized activities. For Noë, the organized activities (i.e., social practices) found across the arts possess ways for us to understand how we come to be through the usage of art’s “strange tools.” Why “strange”? Strange because the tools we habitually use (e.g., music, musical instruments, etc.) let us “know ourselves better”; they do their best work “where, as a matter of habit, we find ourselves” (Noë, 2018, p. 35). Such self-finding, according to Noë, is a “phenomenon of entanglement” where “what we do” and “what we use” and “how we use ‘tools’ of all kinds” bring about “who we are” and “what matters most” to us.^4^

Fundamentally, then, instrumental musicing is something worth doing for the *sake of the self* and others (Elliott and Silverman, 2015), and because of this—note: there is both subjective and objective “value”— instrumental musicing provides a potential vehicle for meaningfulness. As stated in *Music Matters*:

the internal goods and values of musicing are not abstractions. Through the progressive development of musical understanding with musical and educative teachers and facilitators, all students can achieve human flourishing, communal well-being, an empathetic sense of self-and-other, as well as a sense of meaningfulness, enjoyment, and a creative way of life. (Elliott and Silverman, 2015, p. 280)

## Author Contributions

The author confirms being the sole contributor of this work and has approved it for publication.

## Conflict of Interest

The author declares that the research was conducted in the absence of any commercial or financial relationships that could be construed as a potential conflict of interest.

## Footnotes

1 The word “transaction”—which denotes the ways subjects/objects come together and are, not only fused, but are changed due to their coming together—here is taken from Dewey and Bentley’s (1949) *Knowing and the Known*.

2 I am grateful to a reviewer of this paper who pointed out that, while Clark’s work does presage 4E conceptions of being in/of the world, “his theory of extended cognition”—which is primarily a “computational model of human cognition”—seems to run contrary to many of the foundational principles of the majority of the scholars explored throughout this paper (e.g., Varela et al., 1991; Thompson, 2007; Gallagher, 2017).

3 Interestingly, when a musician borrows an instrument that does not belong to her, a period of embodied “adjustment” is necessary. This is why some pianists—like Vladimir Horowitz—travel with their own pianos. It is difficult to gauge the differences among instruments’ “handling.”

4 Again, I am grateful to the reviewer who pointed out that, in many ways, “meaningfulness” is similar to Noë’s (2015) work.
